# A Framework of All Discovered Immunological Pathways and Their Roles for Four Specific Types of Pathogens and Hypersensitivities

**DOI:** 10.3389/fimmu.2020.01992

**Published:** 2020-08-07

**Authors:** Wan-Chung Hu

**Affiliations:** Department of Clinical Pathology, Taipei Tzu Chi Hospital, Buddhist Tzu Chi Medical Foundation, New Taipei City, Taiwan

**Keywords:** Th1/2, Th3, Tr1, Th17, Th9, Th22, Treg, Tfh

## Background of Host Immune Responses

**Figure 1:**
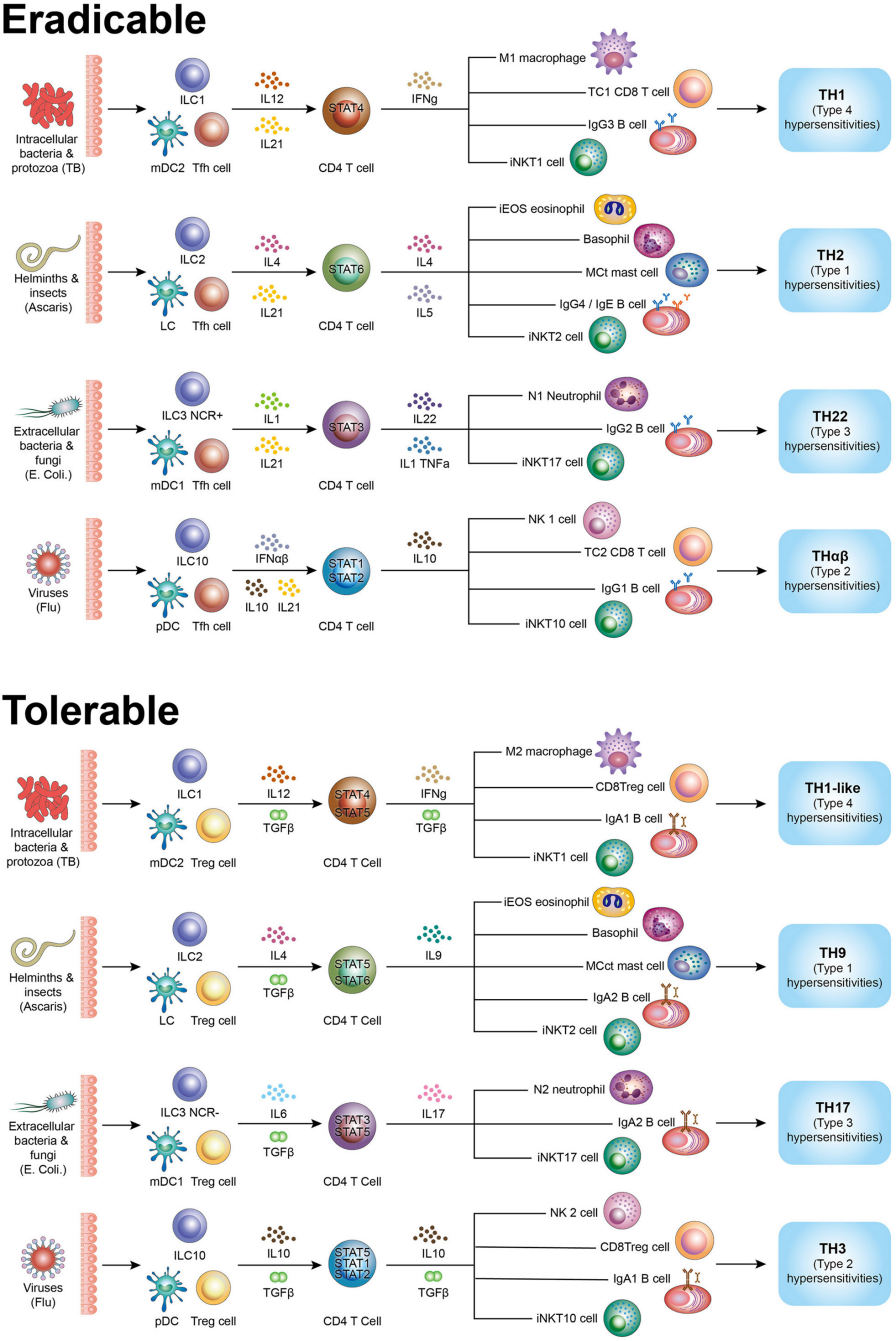
Summary figure of host immunological pathways. Tfh side (follicular help T cell) initiates initiatory immunity and the Treg side (regulatory T cells) initiates regulatory immunity. Eradicable immune responses include TH1, TH2, TH17, and THαβ. Tolerable immune responses include TH1-like (Th1L), TH9, TH22, and TH3. The picture shows all effector cells associated the types of host immune responses. TH1/TH1-like are immune responses against intracellular bacteria/protozoa and are related to type 4 hypersensitivities. TH2/TH9 are immune responses against helminths and are related to type 1 hypersensitivities. TH17/TH22 are immune responses against extracellular bacteria/fungi and are related to type 3 hypersensitivities. THαβ/TH3 are immune responses against viruses and are related to type 2 hypersensitivities.

Although a number of host immunological pathways have been discovered including the traditional TH1/TH2, TH3, TH17, TH22, Tfh, Treg, TH9, and Tr1 (THαβ) pathways, they are not logically organized. In this article, I have described a detailed and complete picture of the host immunological pathways (Figure 1).

The traditional TH1/TH2 paradigm was proposed by Mosmann in 1986 (1). TH1 responses were considered to provide host immunity against intracellular pathogens such as viruses, intracellular bacteria, and protozoa whereas TH2 responses were considered to provide host immunity against multicellular parasites (helminths). In my PhD thesis, I proposed a new THαβ immunological pathway against viruses, derived from the traditional TH1 responses (2), which is associated with intracellular bacteria and protozoa. The TH3 and Tr1 immunological pathways were identified after TH1 and TH2 (3, 4). Additional immune responses including the TH17, TH22, Tfh, Treg, and TH1-like immunological pathways have been discovered recently (5–7).

### Initiatory Immune Response

Follicular helper T cells (Tfh) are considered as the key helper cells for B-cell germinal centers in lymph nodes and are characterized as IL-21-producing T cells (8). Follicular dendritic cells (CD14+) are antigen presenting cells (9), whereas lymphoid tissue inducer cells (LTi) are the innate lymphoid cells for Tfh (10). BCL6 is a key transcription factor in Tfh development (11). TGF-β induced by a STAT5 signal can constrain the differentiation of IL-21-producing helper T cells (12). IL-21 production is related to STAT1and STAT3 activation. IL-21 production is also related to STAT5 activation because immunosuppressive prolactin can cause STAT5a to suppress BCL6 expression (13). In contrast, STAT5b can upregulate BCL6 (14). STAT5a and STAT5b have distinct target genes in immune responses (15), and STAT5b is the transcription factor that induces Tfh. Tfh can induce B-cells to produce IgM antibodies and IL-21 produced by Tfh facilitates B cell isotype switching to IgG (16, 17). Besides the protein antigen recognized by B cells and T cells, natural killer T (NKT) cells also recognize lipid antigens. The subtype iNKTfh plays a role in Tfh responses (18). Thus, T lymphocytes are the first to initiate adaptive host immunity (19–21), wherein different STAT proteins regulate different immunological pathways. If the infection tends to be eradicable, then the host immunological pathways mentioned in the following sections are generated along with other cytokines.

### Eradicable Immune Responses

TH1 immune responses are driven by IL-12 and are induced against intracellular bacteria or protozoa (22). Type 2 myeloid dendritic cells (CD141+ mDC2) are the antigen presenting cells in the TH1 response (9). The main TH1 effector cells are stimulatory macrophages (M1), IFN-γ-secreting cytotoxic CD8 T cells (CD28+ Tc1), IFN-γ-secreting CD4 T cells, iNKT1 cells, and IgG3-producing B-cells (23, 24). Initiation of eradicable immunity also requires innate lymphoid cells to produce the initial cytokines that drive different immunological pathways. For TH1 immune responses, the key innate lymphoid cells are ILC1 (25). STAT4 is the key transcription factor for TH1 immunity but T-bet also plays a vital role. TH1 responses against self-antigens present as type 4 delayed-type hypersensitivity, such as type 1 diabetes mellitus or Crohn's disease (26).

TH2 immune responses are driven by IL-4 and is induced against extracellular parasites (helminths) (27). The antigen presenting cells in TH2 immune responses are Langerhans cells (CD1a+) (9, 28). The main TH2 effector cells are eosinophils (iEOS), basophils/pro-inflammatory mast cells (MCt, mast cell tryptase), IL-4-/IL-5-secreting CD4 T cells, iNKT2 cells, ILC2, and IgG4/IgE-producing B-cells (29). IgG4 activates eosinophils, and IgE activates mast cells, as in acute anaphylaxis (30). IgG4–eosinophils function to activate eosinophil-mediated cellular immunity against parasites or insects, whereas IgE–mast cells act to expel helminths or insects through a physiological mechanism. Mast cells activated by IgE can release histamine, which causes bronchoconstriction, vomiting/nausea, rhinorrhea, skin itching, gastric acidification, increased local vascular permeability, or increased bowel movement. These actions can all help to physiologically expel helminths or insects. The key transcription factor in TH2 response is STAT6 and GATA3 also plays a vital role in the TH2 immunological pathway. TH2 responses against self-antigens present as type 1 immediate allergy, such as food/drug allergy, anaphylaxis, or urticarial (31).

THαβ is distinct from traditional TH1 immune responses (2). THαβ cells are induced against viruses and were previously called as Tr1 cells (4, 32). THαβ immune responses are driven by IFNα/β or IL-10. The antigen presenting cells for THαβ responses are plasmacytoid dendritic cells (pDC) (9). The main effector cells of THαβ immune responses are IL-10-producing stimulatory NK cells (CD56–CD16 + NK1 cells), IL-10/IL-27-secreting CD4 T cells, IL-10-secreting cytotoxic CD8 T cells (CD28+ Tc2), iNKT10 cells, ILC10, and IgG1-producing B-cells (23, 33–35). The CD27 molecule is important for antiviral immunity. The key transcription factors for THαβ response are STAT1 and STAT2 (36). THαβ immune responses against self-antigens present as type 2 antibody-dependent cytotoxic hypersensitivity, such as the acute stage of myasthenia gravis or Graves' disease (37). IL-10 is not merely an immunosuppressive cytokine; it can also have potent stimulatory effects on NK cells, cytotoxic T lymphocytes (CTLs), and B-cells.

TH22 responses are part of the host innate immunity against extracellular bacteria and fungi (38). TH22 response is driven by IL-6 or TNFα (39). The antigen presenting cells in TH22 immune responses are type 1 myeloid dendritic cells (CD1c+ mDC1) (9). The main TH22 effector cells are neutrophils (N1), IL-22-secreting CD4 T cells, iNKT17 cells, ILC3(NCR+), and IgG2-producing B-cells (6, 40). The key TH22 transcription factor is STAT3; AP1 and CEBP are also important transcription factors. TGF-β can suppress IL-22 to skew the TH22 immune response toward TH17 (41). TH22 responses against self-antigens present as type 3 immune-complex and complement-mediated hypersensitivity, such as the Arthus reaction or rheumatoid arthritis (42). The host immunological pathways induced are mainly decided by the extracellular or intracellular location of protozoa or fungi.

Four IgG subtypes fit the four types of acute immunological pathways. Murine IgG antibodies also have four subclasses and are correlated with human IgG subtypes as follows: Human IgG1<->Murine IgG2a; Human IgG2<->Murine IgG3; Human IgG3<->Murine IgG2b; and Human IgG4<->Murine IgG1 (43). hIgG1/mIgG2a function against viral antigens; hIgG2/mIgG3 function against bacterial antigen, especially polysaccharides; hIgG3/mIgG2b act against intracellular bacteria; and hIgG4/mIgG1 act against parasite antigens (44–46). Notably, the immune response against fungi or protozoa is mainly based on their intracellular or extracellular location. Extracellular fungi such as *Candida* spp or *Aspergillus* spp usually trigger TH22 immune responses, whereas intracellular fungi such as *Histoplasma* spp. trigger TH1 responses.

#### Regulatory Immune Responses

Tregs are the host immune inhibitory cells (47) driven by IL-2 and TGF-β. Regulatory dendritic cells (DCreg) are the antigen presenting cells for Tregs (48). Regulatory innate lymphoid cells (ILCreg) are the initial helpers for Treg production (49). The main effector cells for Tregs are the TGF-β-producing CD4 T cells, FOXP3 regulatory iNKT cells, and IgA-producing B-cells (50). STAT5, especially STAT5a is the key transcription factor for the Treg pathway. However, both STAT5a and STAT5b play non-redundant roles in Treg generation (51). They may first act sequentially with STAT5b activation in Tfh signaling. Combined signaling STAT5b and STAT5a induces Treg generation. The combination of Tregs and the aforementioned four immunological pathways are important to shift adaptive immunity to tolerable immunity. During initial infection, acute-stage fierce cytokines can rapidly kill pathogens and infected cells or tissues. However, if the pathogen infects numerous cells in a tissue such as the liver, killing the infected cells will completely destroy the organ (52). Thus, a regulatory T cell STAT5 signal combined with TH1/TH2/TH22/THαβ will allow the generation of CD4 T cells with less fierce inflammatory cytokines (51). TH1-like/TH9/TH17/TH3 immunological pathways are generated during chronic infection. IgA1 and IgA2 are the two types of IgA antibodies, with IgA1 being dominant in the serum, whereas IgA2 is dominant in the mucosa. TGF-β can induce IgA1 or IgA2 depending on the lymphoid follicle location (53). In the Gut-associated lymphoid tissues (GALTs) or the Peyer's patches, IgA2 is the dominant IgA antibody produced in the gastrointestinal mucosa. In the lymph nodes at other body locations, IgA1 is the dominant IgA antibody produced. However, IgA1 is specifically related to viral protein antigens, whereas IgA2 is especially related to bacterial antigens such as LPS. The heavy-chain locus sequence of B-cell antibodies on the human chromosome 14 is IgM, IgD, IgG3, IgG1, IgA1, IgG2, IgG4, IgE, and IgA2. B-cells co-express IgM and IgD. IgG3, IgG1, and IgA1 comprise the first group for cellular immunity, whereas IgG2, IgG4, IgE, and IgA2 can be considered as the second group for humoral immunity. The gene sequence order is important as it affects the time sequence of the isotype switch.

#### Tolerable Immune Responses

TH1-like cells (non-classic TH1) are initiated by TGF-β (STAT5 signaling) and IFN-γ (STAT4 signaling). TH1-like cells with Foxp3+ regulatory characteristics have been identified (7). TH1 helper cells and TH1-like cells are closely related (54). TH1-like cells are induced in chronic TH1 immune responses. Thus, these cells may be related to chronic inflammation such as long-term tuberculosis or leishmania infection (55). The effector cells of TH1-like immune responses include suppressive macrophages (M2), ILC1, suppressive CD8 T cells (CD28-CD8+Treg), IgA1-producing B-cells, iNKT1 cells, and IFN-γ-/TGF-β-producing CD4 T cells (24, 40, 56). The TH1-like response induces type 4 delayed-type hypersensitivity, such as Crohn's disease (26).

TH9 cells are driven by IL-4 (STAT6 signaling) combined with TGF-β (STAT5 signaling) (57–59). Thus, TH9 cells are closely related to the TH2 immunological pathway in parasite immunity (60). The cells are characterized as IL-9-secreting CD4 T cell. TH9 cells are important under a chronic allergic condition such as asthma. Thus, TH9 helper cells are chronic T helper cells related to TH2 immune response. The effector cells of TH9 immunity include regulatory eosinophils, basophils/profibrotic mast cells (MCct, mast cell chymase, and tryptase), ILC2, IL-9-producing CD4 T cells, iNKT2 cells, and IgA2-producing B-cells (40, 61). TH9 immune responses present as type 1 allergy, including asthma (29).

TH17 cells are driven by IL-6/IL-1 combined with TGF-β (5). Thus, TH17 cells are closely related to the TH22 immunological pathway against extracellular bacteria and fungi (62). TH17 cells are characterized as IL-17-secreting CD4 T cells. TH17 cells are important in chronic immune-complex-mediated diseases such as rheumatic arthritis. The TH17 helper cell is the chronic T helper cell related to TH22 immunity. TGF-β with STAT5 can suppress the acute IL-22-producing cells and enhance the chronic IL-17-producing cells (41). Owing to the role of TGF-β in TH17 immunity, regulatory IL-17-producing cells have been noted. The effector cells of TH17 immunity include regulatory neutrophils (N2), ILC3(NCR–), IL-17-producing CD4 T cells, iNKT17 cells, and IgA2-producing B-cells (40, 63). TH17 immunity presents as type 3 immune-complex hypersensitivity, including ulcerative colitis or rheumatoid arthritis (42).

TH3 cells are driven by IL-10 and TGF-β (64, 65). Thus, TH3 cells are closely related to the THαβ immunological pathway against viruses (66). These cells also produce IL-10 and TGF-β. Thus, TH3 helper cells are important for chronic antibody-dependent cellular cytotoxic hypersensitivity. TH3 cells are the chronic helper T cells corresponding to THαβ helper cells. The TH3 immune effector cells include IL-13-producing regulatory NK cells (CD56 + CD16–NK2 cells), ILC10, IL-10- and TGF-β-secreting CD4 T cells, suppressive CD8 T cells (CD28-CD8+ Treg), iNKT10 cells, and IgA1-producing B-cells (34, 35, 56, 67, 68). IgA1 of TH3 immune responses is produced in the serum and acts against viral protein antigens. TH3 immune responses induce type 2 antibody-dependent cytotoxic hypersensitivity, including the chronic stage of Systemic Lupus Erythematosus (SLE) (69).

## Conclusions

The summary diagram includes complete picture of the 4 × 2 + 2 immunological pathways (Table 1). The TH1, TH2, TH22, and THαβ eradicable immune responses correspond with the TH1-like, TH9, TH17, and TH3 tolerable immune responses, respectively, and match the four types of hypersensitivities. The detailed immune responses against different pathogens and in allergy/hypersensitivity can thus be understood clearly. This framework can provide new insights for therapeutic agent development for the four types of pathogens and hypersensitivities.

**Table 1:** Summary of host immunological pathways.

**Immune pathways**	**Driven cytokines, ILCs, DC**	**Transcription factors**	**Effector cells**	**CD4 T cells**	**B cells**	**NKT cells**	**Pathogen/** **pathogenesis**	**Autoimmune**
**Initiatory**								
Tfh	IL-21, FDC, LTi	STAT1, STAT3, STAT5B		IL-21 CD4 T cells	IgM/G B cells	iNKTfh	Acute infection	
**Eradicable immunities**								
TH1	IL-12, ILC1, mDC2	STAT4	Macrophages (M1), CTL (Tc1)	IFN-γ CD4 T cells	IgG3 B cells	iNKT1	Intracellular bacteria and protozoa	Type 4 DTH
TH2	IL-4, ILC2, LC	STAT6	Eosinophils (iEOS), basophils, mast cells (MCt)	IL-4, IL-5, CD4 T cells	IgG4/IgE B cells	iNKT2	Helminths and insects	Type 1 allergy
TH22	IL-1, mDC1, ILC3 NCR+	STAT3	Neutrophils (N1)	IL-1, TNFα, IL-22 CD4 T cells	IgG2 B cells	iNKT17	Extracellular bacteria and fungi	Type 3 Immune complex
THαβ	IL-10, pDC, IFNα, ILC10	STAT1, STAT2	NK cells (NK1), CTL (Tc2)	IL-10 CD4 T cells	IgG1 B cells	iNKT10	Viruses	Type 2 ADCC
Immune pathways	Driven cytokines, ILCs	Transcription factors	Effector cells	CD4 T cells	B cells	NKT cells	Pathogen/ pathogenesis	Hypersensitivities
**Regulatory**								
Treg	TGF-β, DCreg, ILCreg	STAT5A, STAT5B		TGF-β CD4 T cells	IgA B cells	iNKT1	Chronic infection	
**Tolerable immunities**								
TH1 like	IL-12, TGF-β, ILC1	STAT4, STAT5	Macrophages (M2), CD8 Tregs	IFN-γ/TGF-β CD4 T cells	IgA1 B cells	iNKT2	Intracellular bacteria and protozoa	Type 4 DTH
TH9	IL-4, TGF-β, ILC2	STAT6, STAT5	Eosinophils(rEOS), basophils, mast cells (MCct)	IL-9 CD4 T cells	IgA2 B cells	iNKT17	Helminths and insects	Type 1 allergy
TH17	IL-6, TGF-β, ILC3 NCR–	STAT3, STAT5	Neutrophils (N2)	IL-17 CD4 T cells	IgA2 B cells	iNKT10	Extracellular bacteria and fungi	Type 3 immune complex
TH3	IL-10, TGF-β, ILC10	STAT1, STAT2, STAT5	NK cells (NK2), CD8 Tregs	IL-10/TGF-β CD4 T cells	IgA1 B cells	iNKTreg	Viruses	Type 2 ADCC

## Author Information

The author, Wan-Chung Hu, graduated as an MD from National Taiwan University. He then completed his Ph.D. in vaccine science from the Department of International Health, Johns Hopkins University, Bloomberg School of Public Health. The author's Ph.D. thesis was based on host immune responses against malarial infection. He conducted a postdoctoral study on cancer immunotherapy at the Genomics Research Center, Academia Sinica, Taiwan. The author completed his PGY training from Mackay Memorial Hospital and Shin-Kong Memorial Hospital in Taiwan. He has worked as a chief resident at the Department of Clinical Pathology, Far Eastern Memorial Hospital, Taiwan (R.O.C.), for resident training. He is currently a physician-scientist and an attending clinical pathologist at the Division of Clinical Pathology of Taipei Tzu Chi Hospital.

## Author Contributions

W-CH solely contributed to the concept, literature search, writing, and final approval of this manuscript.

## Conflict of Interest

The author declares that the research was conducted in the absence of any commercial or financial relationships that could be construed as a potential conflict of interest.
